# False discovery rate control for sparse correlation matrix

**DOI:** 10.1093/bioadv/vbag314

**Published:** 2026-10-19

**Authors:** Arief Gusnanto, Basuki Widodo, Norli Anida Abdullah, Ahmad Zia Ul-Saufie, Lucy F Stead

**Affiliations:** Department of Statistics, University of Leeds, Leeds, LS2 9JT, United Kingdom; Department of Mathematics, Institut Teknologi Sepuluh Nopember, Surabaya 60111, Indonesia; Mathematics Division, Centre for Foundation Studies in Science, Universiti Malaya, Kuala Lumpur 50603, Malaysia; Faculty of Computer and Mathematical Sciences, Universiti Teknologi MARA, Shah Alam 40450, Malaysia; Leeds Institute of Medical Research at St. James’s, University of Leeds, Leeds, LS9 7TF, United Kingdom

## Abstract

**Motivation:**

In genomic data analysis, high-dimensional correlation matrices pose challenges in interpreting complex relationships among genomic features, particularly due to overfitting. Traditional approaches generate sparse correlation matrices by setting correlations below a predetermined threshold to zero, with the threshold typically determined through Frobenius norm minimization via cross-validation.

**Results:**

In this study, we propose a novel alternative thresholding strategy based on an empirical Bayes framework that controls the false discovery rate among nonzero correlations. This method offers two key advantages: first, it is compatible with any correlation measure and, second, it provides an estimation of the expected false positives among nonzero correlations. By formulating the construction of a sparse correlation matrix as both an estimation and an inference problem, our approach enhances interpretability and demonstrates greater sensitivity in retaining true biological correlations than threshold selection based on the minimization of Frobenius norm.

**Availability and implementation:**

The relevant R package called scfdr is available from github.com/ariefgusnanto/scfdr

## 1 Introduction

In analysis of gene expression data, identifying groups of genes that exhibit similar expression patterns across samples is critical for many reasons. For example, to understand gene function and regulation, co-expression analysis helps in constructing gene co-expression networks, which illustrate how genes interact and regulate each other (Langfelder and Horvath 2008). By identifying groups of co-expressed genes, researchers can also infer the function of unknown genes based on the known functions of their co-expressed partners (Gaiteri *et al.* 2014). In cancer research, co-expression analysis can reveal gene modules and network patterns associated with tumour progression and patient prognosis (Ma *et al.* 2010, Yang *et al.* 2014). Analysing how gene expression changes in response to drug treatments also helps in understanding the mechanisms of action and can guide the development of more effective therapies (Garnett *et al.* 2012). Furthermore, co-expression analysis can help identify gene modules and hub genes associated with disease prognosis and clinically relevant stratification, thereby supporting downstream biomarker discovery and more personalized analysis (Ma *et al.* 2010, Shi *et al.* 2017).

For those purposes, a correlation matrix has been instrumental in measuring the co-expression between genes. More broadly, measures of statistical association between genes underpin several frameworks widely used in gene-expression studies, including cluster analysis, principal component analysis, linear discriminant analysis, co-expression or interaction-network reconstruction (Jiang, *et al.* 2004, Zhang and Horvath 2005, Paliwal and Sharma 2010, Serra *et al.* 2018), and related graphical-model approaches such as partial-correlation Gaussian graphical models and Bayesian networks incorporating biological prior knowledge (Werhli and Husmeier 2007, Bernal *et al.* 2019). However, this approach has significant limitations, particularly in the context of high-dimensional genomic data where the number of variables far exceeds the number of observations. The first immediate challenge is overfitting: in high-dimensional settings, empirical correlation matrices can capture spurious associations that reflect sampling variation and noise rather than genuine biological relationships (Bickel and Levina 2008, Serra *et al.* 2018). Second, the standard (dense) correlation matrices are difficult to interpret because almost all (if not all) of the entries are nonzero. For example, for data with 20 000 genes, we need to interpret almost 200 million correlations between pairs of genes. Identifying meaningful gene–gene associations among such a large number of nonzero correlations can therefore require additional postprocessing, such as network construction, topological-overlap transformation, or resampling-based inference (Luo *et al.* 2007, Cai and Liu 2016, Colby *et al.* 2018, Voigt and Almaas 2019). These procedures introduce further modelling and tuning choices and may require substantial additional computation merely to obtain an interpretable representation of the original correlation matrix.

In recent years, there has been a growing recognition of the advantages of estimating sparse correlation matrices, which assume that many of the true correlations are zero. By imposing sparsity, we can mitigate the risk of overfitting and focus only on the most significant correlations that lead to more robust and interpretable results (Meinshausen and Bühlmann 2006). This simplification is particularly valuable in uncovering biologically meaningful patterns (Friedman *et al.* 2008). Sparse correlation matrices are often constructed by setting correlations with absolute value below a threshold to zero (‘hard thresholding’), in contrast to ‘soft thresholding’, which shrinks correlations toward zero while retaining all entries (Langfelder and Horvath 2008). More generally, hard-thresholding estimators have a substantial literature in sparse covariance and correlation estimation, with threshold selection often guided by a matrix loss such as the Frobenius norm (FN; Bickel and Levina 2008; Rothman *et al.* 2008, 2009; Jiang 2013). In genomic applications, Serra *et al.* (2018) adapted this strategy to robust correlation estimation for high-dimensional gene expression data.

We find that a threshold selected to minimize global matrix-estimation error (FN) need not be optimal for identifying truly correlated gene pairs. In particular, it may be overly conservative from a discovery perspective and thus reduce sensitivity, especially for moderate but biologically meaningful correlations. In this work, we therefore formulate the threshold selection as an inferential problem and choose the hard threshold by controlling the false discovery rate (FDR) among the retained nonzero correlations. This leads to a sensible interpretation and a straightforward inference on the nonzero correlations. We emphasize that our concern is not with hard thresholding itself; indeed, our proposed method also uses hard thresholding. Rather, the issue is the criterion used to choose the threshold.

The paper is organized as follows. The relevant methodology is described next in Section 2, including the datasets used to illustrate the method. Section 3 shows the results, and the discussion is presented in Section 4. The conclusion is presented in Section 5.

## 2 Methods

### 2.1 Dataset

To illustrate our approach, we consider two gene expression datasets. The first one is from the Cancer Cell Line Encyclopedia (CCLE) project (Barretina *et al.* 2012) that compiled gene expression data (among other types of data) from human cancer cell lines. For our purpose, we focus on glioma cancer lines. The data were downloaded from gene expression omnibus with accession number GSE36133. The data consist of 43 arrays with 54 675 probesets. We pre-processed the data using robust multiarray analysis (RMA) for normalization (Bolstad *et al.* 2003, Irizarry *et al.* 2003). After this step, we only consider 13 044 probesets that are ‘present’ in all arrays (patients) according to the Microarray Suite 5 algorithm (McClintick and Edenberg 2006) for further analysis.

The second dataset is the glioblastoma (GBM) gene expression data from The Cancer Genome Atlas (TCGA; Cancer Genome Atlas Research Network 2012, 2014; Brennan *et al.* 2013), under the Genomic Data Commons (GDC; Grossman *et al.* 2016). The data were downloaded from Xena Functional Genomics Explorer in the University of California, Santa Cruz (Goldman *et al.* 2020). We consider the Fragments Per Kilobase of transcript per Million mapped reads with upper quantile normalization (FPKM-UQ) for our illustration. In the data, we have 173 patients with expressions from 18 467 genes.

These normalization choices follow standard pre-processing conventions for the respective platforms. We used RMA for the Affymetrix microarray data because it provides established background correction, normalization, and summarization (Bolstad *et al.* 2003, Irizarry *et al.* 2003), whereas for the TCGA RNA-seq data, we used the FPKM-UQ values as provided by the Xena/GDC resource. Since the two datasets are analysed separately and serve only to illustrate the proposed thresholding strategy, rather than for direct cross-platform comparison, these choices are not expected to affect the methodological conclusions. We nevertheless note that normalization may influence the induced correlation structure and should therefore be considered when interpreting dataset-specific results.

### 2.2 Correlation

We first introduce the notation we shall use in this study. For this purpose, and without loss of generality, we consider the case of gene expression data although our approach can generally be implemented in any other genomic data where computing correlation between genomic features is relevant and, indeed, biologically meaningful. Let $x_{ij}$ be the expression of gene *j* in individual *i*, i=1,2,…,n and j=1,2,…,p. We assume that the data have been properly normalized prior to analysis (for the datasets considered here, the normalization procedures are described in Section 2.1), since otherwise the resulting correlations may reflect technical artefacts rather than biologically meaningful association. We denote the sample (observed) correlation between a pair of genes *j* and $j^{\prime }$, $j\neq j^{\prime }$ and $j^{\prime }=1,2,\ldots ,p$, as $r_{jj^{\prime }}$. For the correlation measure, we consider the robust correlation based on median absolute deviation (MAD) used by Serra *et al.* (2018), which was originally proposed in Pasman and Shevlyakov (1987). For completeness, we also include some existing well-known correlation measures in our computational package, such as Pearson correlation, Spearman correlation, and Kendall correlation. However, our study does not aim to compare different correlation measures, nor propose a new one. The choice of which correlation measure to consider depends on the characteristics of the data, such as robustness to noise and outlying observations, as discussed further in Section 4. Our approach in this study does not depend on which correlation measure used, which is an advantage of the local FDR control discussed in the next section.

The robust MAD correlation can be described as follows. For a pair of genes *j* and $j^{\prime }$, $j\neq j^{\prime }$, robust MAD correlation is defined by


$$r_{jj^{\prime }}^{\text{MAD}}=\frac{{\text{MAD}}^{2}(u)-{\text{MAD}}^{2}(v)}{{\text{MAD}}^{2}(u)+{\text{MAD}}^{2}(v)},$$

where

-
$u=\frac{x_{j}-\text{median}(x_{j})}{\sqrt{2}\text{MAD}(x_{j})}+\frac{x_{j^{\prime }}-\text{median}(x_{j^{\prime }})}{\sqrt{2}\text{MAD}(x_{j^{\prime }})}$

-
$v=\frac{x_{j}-\text{median}(x_{j})}{\sqrt{2}\text{MAD}(x_{j})}-\frac{x_{j^{\prime }}-\text{median}(x_{j^{\prime }})}{\sqrt{2}\text{MAD}(x_{j^{\prime }})}$ , and
-
$\text{MAD}(x_{j})=\text{median}\left(\left|x_{ij}-\text{median}(x_{j})\right|\right)$ for i=1,2,…,n

and |·| denotes the modulus or absolute value. We use the robust MAD correlation as a practical default in the analysis because it is robust to outlier and computationally scalable (Serra *et al.* 2018). Genomic data tend to have some characteristics that expose the drawbacks of many well-known correlations, which we will revisit later in the discussion.

### 2.3 Local FDR

We now introduce a more general notation for the correlation statistics described above. Let $Z_{jj^{\prime }}$ denote the random correlation statistic for the pair of genes ($j,j^{\prime }$), with $j<j^{\prime }$, and let $z_{jj^{\prime }}$ denote its observed/realized value. Thus, throughout this section, uppercase letters refer to random variables and lowercase letters to their realized values. For notational simplicity, when no ambiguity arises, we suppress the pair-specific indices and write *Z* for the generic correlation statistic and *z* for its observed value. We consider only the off-diagonal elements of the correlation matrix, giving a total of $n_{q}=p(p-1)/2$ pairwise correlations.

We consider the empirical Bayes formulation (Efron *et al.* 2001, Ploner *et al.* 2006) where we assume that the distribution of the observed correlations, denoted f(z), to follow a mixture distribution


(1) $$f(z)=\pi_{0}f_{0}(z)+(1-\pi_{0})f_{1}(z)$$

where $\pi_{0}$ is the proportion of noncorrelated pairs of genes, $0<\pi_{0}<1$. The densities $f_{0}(z)$ and $f_{1}(z)$ are the distribution of statistics of pairs of genes that are noncorrelated and correlated, respectively. We also follow the convention (Efron *et al.* 2001, Ploner *et al.* 2006) to allow $f(z),f_{0}(z)$ and $f_{1}(z)$ to be nonparametric. In practice, f(z) can be estimating using the kernel density of the observed correlations and $f_{0}(z)$ from permuted data under the null hypothesis that there is no correlation between any pair of genes. The latter will be described in the next section.

The local FDR is calculated as follows:


(2) $$\text{FDR}(z)=P(H_{0}|Z=z)=\pi_{0}\frac{f_{0}(z)}{f(z)}$$

where $H_{0}$ denotes the event that the gene pair is truly uncorrelated. It is therefore interpreted as the posterior probability that a gene pair is truly uncorrelated, conditional on observing correlation statistic Z=z. It can be considered as the expected proportion of false positives if a pair of genes with (observed) correlation Z=z are declared to be correlated. The quantity 1−FDR(z), on the other hand, is interpreted as the posterior probability that pair of genes being correlated given that the correlation between the pair is Z=z.

A useful consequence of this formulation is that the (global) FDR over any rejection region (Benjamini and Hochberg 1995) can be expressed as the conditional mean of the local FDR among the selected correlations (Ploner *et al.* 2006). Let R denote the rejection region, so that a pair of genes is declared correlated whenever Z∈R. Then the global FDR is given by


$$\text{FDR}(R)=P(H_{0}|Z\in R)$$

or, by the law of iterated expectation, this can be written as follows:


(3) $$\text{FDR}(R)=E\left[P(H_{0}|Z)|Z\in R\right]=E\left[\text{fdr}(Z)|Z\in R\right]$$

Since the local FDR at the observed value *z* is given by (2), the above conditional expectation can be written as follows:


(4) $$\text{FDR}(R)=\frac{\int_{R}\text{fdr}(z)f(z)d\;z}{P(Z\in R)}=\frac{\int_{R}\pi_{0}f_{0}(z)dz}{P(Z\in R)}$$

Therefore, the global FDR is simply the average of the local FDRs over the correlations whose observed values fall in the rejection region. In practice, this is estimated by averaging the local FDR values of the retained observed correlations, as described in Section 2.5.

This means that, in declaring whether a pair of genes is correlated or not, the usual control of FDR in estimating a genome-wide correlation matrix can be implemented. For example, consider a situation where we have 15 000 genes in our dataset, which means there are ∼112.5 million correlations between genes. Suppose our approach declares 50 000 pairs of genes to be correlated with (global) FDR 1%. This means that 500 correlations are expected to be false positives. This simple interpretation and statement is a major advantage when we are dealing with correlation of high-dimensional genomic data.

### 2.4 Estimating $f_{0}(z)$ and $\pi_{0}$

Within this empirical Bayes formulation (Efron *et al.* 2001), the estimation of $f_{0}(z)$ and $\pi_{0}$ provides the ingredients required to evaluate the local FDR in (2). As mentioned above, the density of uncorrelated pairs of genes $f_{0}(z)$ is estimated under the null hypothesis that none of the pairs of genes is correlated. Under the null hypothesis that there is no pair of genes that are correlated, we perform the following steps:

1. We randomly select a pair of genes, e.g. $x_{m}$ and $x_{m^{\prime }}$, $m,m^{\prime }\in \{1,2,\ldots ,p\}$, $m\neq m^{\prime }$.
2. Within each of $x_{m}$ and $x_{m^{\prime }}$, we independently shuffle the gene expressions for those genes under the null hypothesis. This will create the vectors of (shuffled) gene expressions $x_{m}^{*}$ and $x_{m^{\prime }}^{*}$.
3. Calculate the correlation between $x_{m}^{*}$ and $x_{m^{\prime }}^{*}$, denoted $z^{*}\equiv r(x_{m}^{*},x_{m^{\prime }}^{*})$.
4. We repeat Steps 1–3 above $n_{s}$ times to obtain $n_{s}$ correlations under the null hypothesis. Our experience suggested that $n_{s}=0.04p^{2}$ for p>10 000 is generally sufficient to sustain the mass of $f_{0}(z)$.
5. The density of the uncorrelated pairs of genes $f_{0}(z)$ is therefore estimated as $f(z^{*})$.

We estimate the proportion of the uncorrelated pairs of genes, $\pi_{0}$, by noting that FDR(z) is bounded by one. Therefore,


(5) $$\pi_{0}\leq {\text{min}}_{z}\frac{f(z)}{f_{0}(z)}$$

and we take this upperbound of as an estimate of $\pi_{0}$ (Efron *et al.* 2001). This permutation scheme is used to estimate the *marginal* null distribution of the chosen correlation statistic for a single gene pair: by independently shuffling the two expression vectors, it preserves the marginal distribution of each gene while removing pairwise association under the null (Efron *et al.* 2001). It is not intended to reproduce the full joint dependence structure across all pairwise correlations, which may be induced by shared genes or broader latent structure (Pawitan *et al.* 2006).

### 2.5 Threshold for sparse correlation matrix construction

The key step in the construction of a sparse correlation matrix with hard thresholding is to identify the threshold *h*, below which the correlations will be set to zero. Consider the case of correlations between genes *j* and $j^{\prime }$, $z_{jj^{\prime }}$, for $j\neq j^{\prime }$. To create a sparse correlation matrix $Z^{s}$ with elements $z_{jj^{\prime }}^{s}$, we perform the thresholding on $z_{jj^{\prime }}$ such that


(6) $$z_{jj^{\prime }}^{s}=z_{jj^{\prime }}I(|z_{jj^{\prime }}|\geq h)$$

where I(·) is the indicator function, which equals to one if the expression within the brackets is correct and zero otherwise.

The threshold *h* is chosen such that the (global) FDR of the correlations that pass the threshold is less than or equal to a prespecified value δ. In other words, we set *h* such that


(7) FDR(R(h))≡E(fdr(z)|R(h))≤δ

In practice, we rank the observed correlations by decreasing absolute magnitude and, for the top *t* correlations, estimate the global FDR by the average of their corresponding local FDR values. The threshold *h* is then chosen as the largest value for which this running average does not exceed the prespecified level δ (further details can be found in the Supplementary Material, available as supplementary data at *Bioinformatics Advances* online). For the choice of δ, our experience suggested that a more stringent FDR control is generally a sensible approach for a first investigation before relaxing the control if necessary. Since we are dealing with a large number of correlations, proportional to $p^{2}$ and can be in hundreds of millions, we found that setting the FDR control at 0.001 or 0.002 is a sensible first choice.

It is important to note that the construction of a sparse correlation matrix here does not necessarily produce a positive definite matrix, as rightly noted by Serra *et al.* (2018). The standard (dense) correlation matrix itself is not positive definite when n<p, which is generally the case in genomic data. If the positive definiteness is absolutely needed, then we can resort to the nearest correlation matrix algorithm (Higham 2002, Serra *et al.* 2018). However, forcing the positive definitiveness does not guarantee that the sparseness is still retained (Serra *et al.* 2018).

### 2.6 Simulation study

To understand the working characteristics of the proposed method to produce a sparse correlation matrix, we perform a simulation study. In the simulation, we vary the simulated correlation between some genes and record the sensitivity and specificity of the produced sparse correlation matrix. The sensitivity is defined as how many truly nonzero correlations that are retained as nonzero (or, survive the thresholding), rather than the power of a formal sample-size-calibrated hypothesis test. Similarly, specificity is defined as how many truly zero correlations that are set to zero in the thresholding stage. In addition, we also calculate the FN between the produced sparse correlation matrix with the true correlation matrix.

We compare our proposed method with the robust sparse correlation (RSC) method (Serra *et al.* 2018) to construct a sparse correlation matrix. Both methods use the robust MAD as a correlation measure and hard thresholding to create a sparse correlation matrix. However, the RSC method identifies the threshold by minimizing the FN while our proposed method identifies the threshold by controlling FDR on the retained nonzero correlation. To illustrate the generalizability of the proposed method, we also perform the simulation study using the Spearman correlation coefficient, and the results are presented in the Supplementary Material, available as supplementary data at *Bioinformatics Advances* online.

The simulated data are generated as follows. A matrix of gene expression data with a dimension of n=100 by p=1000 are generated as multivariate normal with mean μ with correlation matrix Ψ; equivalently, Ψ is also the covariance matrix because all marginal variances are fixed at one throughout the simulation. The mean vector μ has elements that range from 0.5 to 1.5. The covariance/correlation matrix Ψ is of size 1000×1000, with the following structure:


(8) $$\begin{array}{c} \Psi =\left[\begin{array}{ccccc} \Psi_{11} & 0 & 0 & \cdots & 0\\ 0 & \Psi_{22} & 0 & \cdots & 0\\ 0 & 0 & I & \cdots & 0\\ \vdots & \vdots & \vdots & \ddots & \vdots \\ 0 & 0 & 0 & \cdots & I \end{array}\right] \end{array}$$

where *I* is the identify (sub-)matrix. This indicates that there are two correlation blocks that, in genomic application, can be considered as networks. Outside those two correlation blocks, the variables are independent. The submatrix $\Psi_{kk}$, k=1,2, is defined as follows:


(9) $$\begin{array}{c} \Psi_{kk}=\left[\begin{array}{cccc} 1 & \rho & \cdots & \rho \\ \rho & 1 & \cdots & \rho \\ \vdots & \vdots & \ddots & \vdots \\ \rho & \rho & \cdots & 1 \end{array}\right] \end{array}$$

where ρ is the true correlation between pairs of variables in the same correlation block.

The simulation settings are set as follows:

- True correlation ρ is set at different values: 0.3, 0.5, 0.7, and 0.9.
- The size of $\Psi_{kk}$ is set at 25×25 and 50×50.
- For the proposed method, the level of FDR controlled is set to range from 0.001 to 0.05.

For each combination of settings, we generate 1000 simulated datasets. The purpose of varying the different parameter settings is to understand how the sensitivity, specificity, and FN change as those values change or increase/decrease. The range of ρ, in particular, is considered to represent weak to strong correlation, while the different levels of FDR control enable us to understand the impact of different levels of stringency in the control.

This simulation study is intended primarily to assess how well the proposed thresholding rule recovers the underlying sparse correlation structure. We therefore summarize performance using sensitivity, specificity, and FN, which quantify recovery of truly nonzero correlations, removal of true zeros, and overall matrix accuracy, respectively. This choice is deliberate, because the method is used here as a threshold-selection rule for constructing a sparse correlation matrix, so the central question is support recovery. In contrast, the false discovery proportion (FDP) is a replicate-specific random quantity, whereas the FDR is its expectation over repeated experiments (Benjamini and Hochberg 1995, Genovese and Wasserman 2002). In highly sparse settings such as those considered here with $1-\pi_{0}=0.0006$, the realized FDP can vary markedly across replicates and is therefore a less stable summary of structural recovery than sensitivity and specificity (Meinshausen and Bühlmann 2006, Pawitan *et al.* 2006).

## 3 Results

### 3.1 Simulated data

The results of the simulation are presented in Table 1 for the robust MAD correlation; the simulation results using the Spearman correlation are presented in the Supplementary Material, available as supplementary data at *Bioinformatics Advances* online. Overall, the table indicates that the proposed method has better operating characteristics than that of RSC (Serra *et al.* 2018) at different levels of FDR control. This is indicated with higher sensitivity, while maintaining high specificity and low FN. This advantage is more substantial when the true correlation is in the range 0.3–0.7. When the true correlation is 0.9, the table indicates that both methods have equally good performance. This is not surprising because the high correlation is relatively easy to be detected and survive the thresholding. In the case of low correlation (ρ=0.3), a difficult case, we can see that RSC has no sensitivity while our approach manages to have some (albeit low) sensitivity. This situation improves further as we relax the FDR control and our proposed method still indicates an advantage in sensitivity.

**Table 1:** Sensitivity, specificity, and FN of sparse correlation matrix from 1000 simulated data under different simulation settings using the robust MAD correlation.^a^

ρ	FDR	$\Psi_{kk}:50\times 50$			$\Psi_{kk}:25\times 25$		
		Sens.	Spec.	FN	Sens.	Spec.	FN
**0.3**	RSC	0.000	1.000	0.000	0.000	1.000	0.000
	0.001	0.016	0.995	0.001	0.025	0.985	0.001
	0.002	0.019	1.000	0.000	0.018	1.000	0.000
	0.003	0.027	1.000	0.001	0.026	1.000	0.000
	0.005	0.040	1.000	0.001	0.039	1.000	0.000
	0.010	0.070	0.999	0.001	0.067	0.999	0.000
	0.015	0.095	0.998	0.001	0.091	0.998	0.001
	0.020	0.117	0.997	0.001	0.113	0.997	0.001
	0.050	0.223	0.990	0.002	0.219	0.991	0.002
**0.5**	RSC	0.162	1.000	0.001	0.046	1.000	0.000
	0.001	0.225	1.000	0.001	0.213	1.000	0.000
	0.002	0.305	1.000	0.001	0.285	1.000	0.000
	0.003	0.360	1.000	0.001	0.334	1.000	0.000
	0.005	0.433	0.999	0.001	0.400	0.999	0.000
	0.010	0.535	0.998	0.001	0.499	0.999	0.000
	0.015	0.595	0.997	0.001	0.560	0.998	0.001
	0.020	0.638	0.996	0.001	0.605	0.997	0.001
	0.050	0.770	0.988	0.003	0.752	0.990	0.002
**0.7**	RSC	0.893	1.000	0.000	0.823	1.000	0.000
	0.001	0.930	1.000	0.000	0.913	1.000	0.000
	0.002	0.956	0.999	0.000	0.939	1.000	0.000
	0.003	0.966	0.999	0.000	0.951	0.999	0.000
	0.005	0.975	0.998	0.001	0.963	0.999	0.000
	0.010	0.984	0.997	0.001	0.976	0.998	0.000
	0.015	0.988	0.996	0.001	0.983	0.997	0.001
	0.020	0.990	0.994	0.001	0.986	0.996	0.001
	0.050	0.996	0.986	0.003	0.995	0.989	0.002
**0.9**	RSC	1.000	1.000	0.000	1.000	1.000	0.000
	0.001	1.000	0.999	0.000	1.000	1.000	0.000
	0.002	1.000	0.999	0.000	1.000	0.999	0.000
	0.003	1.000	0.999	0.000	1.000	0.999	0.000
	0.005	1.000	0.998	0.001	1.000	0.999	0.000
	0.010	1.000	0.997	0.001	1.000	0.998	0.000
	0.015	1.000	0.995	0.001	1.000	0.997	0.001
	0.020	1.000	0.994	0.001	1.000	0.996	0.001
	0.050	1.000	0.986	0.003	1.000	0.989	0.002

a The FDR is controlled from 0.001 to 0.05, with the ‘RSC’ row corresponds to construction of a sparse correlation matrix by minimizing the FN (Serra *et al.* 2018). The true correlation ρ is set to be from 0.3 to 0.9, and the dimension of the $\Psi_{11}$ and $\Psi_{22}$ submatrix in the true correlation matrix is 50×50 and 25×25 (9). The simulation results using the Spearman correlation are presented in the Supplementary Material, available as supplementary data at *Bioinformatics Advances* online.

### 3.2 Real data

For both real datasets, the primary analysis was carried out using the robust-MAD and Spearman correlations described in Section 2.2, with the sparse correlation matrix constructed by controlling the FDR at 0.001 according to Sections 2.3–2.5; unless otherwise stated, no dataset-specific tuning parameters were introduced. The results of the analysis on the glioma (GSE36133) data using robust MAD correlation are presented in Fig. 1; the results using the Spearman correlation are presented in the Supplementary Material, available as supplementary data at *Bioinformatics Advances* online. The figure (left panel) presents the histogram and density of the observed correlations f(z) and the density of correlations under the null hypothesis of no correlation between genes. The plot shows an excess of variability of the observed correlations (compared to that under the null hypothesis), indicating some correlations are expected to be real. Finally, the figure (right panel) also presents the posterior probability 1−FDR(z). This probability is interpreted as the probability of the corresponding pair of genes to be correlated given Z=z. The plot indicates that as the observed correlation is zero, this probability is also zero. However, as the observed correlations move away from zero in both directions, the posterior probability of true correlation is increasing towards one.

**Figure 1:**
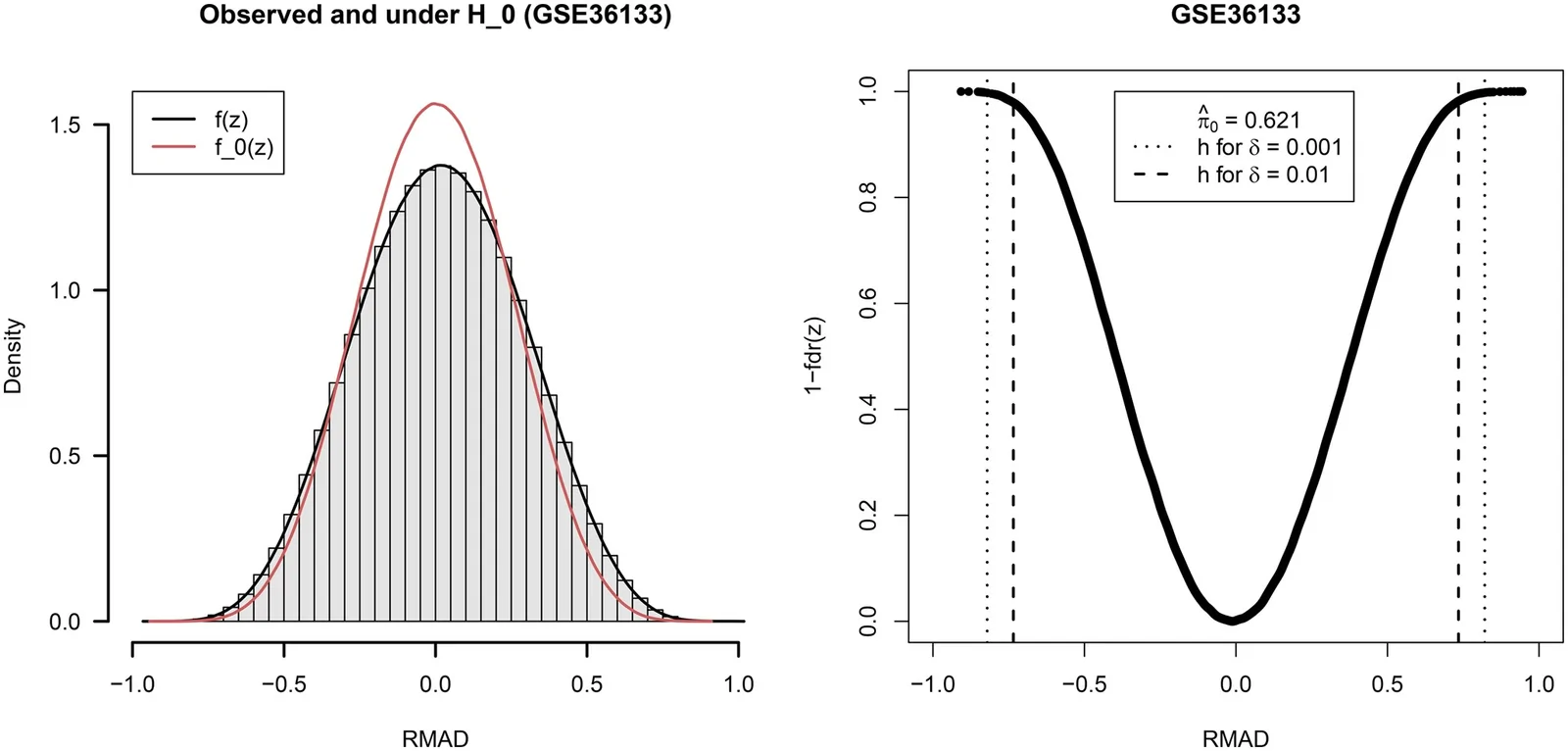
Results from the glioma data (GSE36133) using robust MAD correlation. Left panel: histogram and density of the observed correlations (f(z)), and the density of marginal correlations under the null ($f_{0}(z)$). Right panel: posterior probability of correlation 1−FDR(z) as a function of the observed correlations. The dotted and dashed vertical lines correspond to the threshold *h* when we control FDR at δ=0.001 and δ=0.01, respectively. The estimated proportion of null correlations is ${\hat{\pi }}_{0}=0.621$.

Using FDR control at 0.001, the analysis indicates that the threshold to create a sparse correlation matrix is at 0.821. So, any correlations that are less than this threshold in absolute value is set to zero. This resulted on 99.977% of correlations to be set to zero with only 19 626 correlations (0.023%) to be nonzero in the sparse correlation matrix. With this level of FDR control, we expect that 20 correlations to be false positives. It can be shown that when we relax the FDR to 0.01, the resulting sparse correlation matrix contains >174 000 nonzero correlations, or ∼0.2%. This behaviour is consistent with the simulation study: relaxing the FDR target lowers the threshold *h*, so that more correlations survive thresholding, increasing sensitivity of the sparse solution while controlling the expected proportion of false positives at the chosen level.

To illustrate some correlations from the sparse correlation matrix (with FDR 0.001), we present a circular plot for genes that are correlated with each of NEK2 and CCNA2 as shown in the Supplementary Material, available as supplementary data at *Bioinformatics Advances* online. Both genes are recently been identified to regulate GBM progression by targeting the cell cycle (Zhou *et al.* 2024). In the case of NEK2, the gene is correlated with 16 other genes while in the case of CCNA2, it is correlated with 42 other genes. To illustrate that the proposed method can be used with other correlation measures, we present the analysis results using the Spearman correlation in the Supplementary Material, available as supplementary data at *Bioinformatics Advances* online.

The results of the analysis on the TCGA-GBM data using robust MAD correlation are presented in Fig. 2; the results using the Spearman correlation are presented in the Supplementary Material, available as supplementary data at *Bioinformatics Advances* online. Similar to the previous result on glioma data, the left panel figure presents the histogram and density of the observed correlations f(z) and the density of the correlations under the null $f_{0}(z)$. The left panel figure indicates a substantial excess of variability of the observed correlations (f(z)) compared to that under the null hypothesis ($f_{0}(z)$), indicating many correlations are expected to be real. This not surprising because there are more patients in the TCGA-GBM data than the glioma data, and we expect more power to detect the correlations. Finally, the right panel figure also presents the posterior probability 1−FDR(z). We also have similar indication to Fig. 1 where, as the observed correlations move away from zero in both directions, the posterior probability of true correlation is increasing towards one from zero.

**Figure 2:**
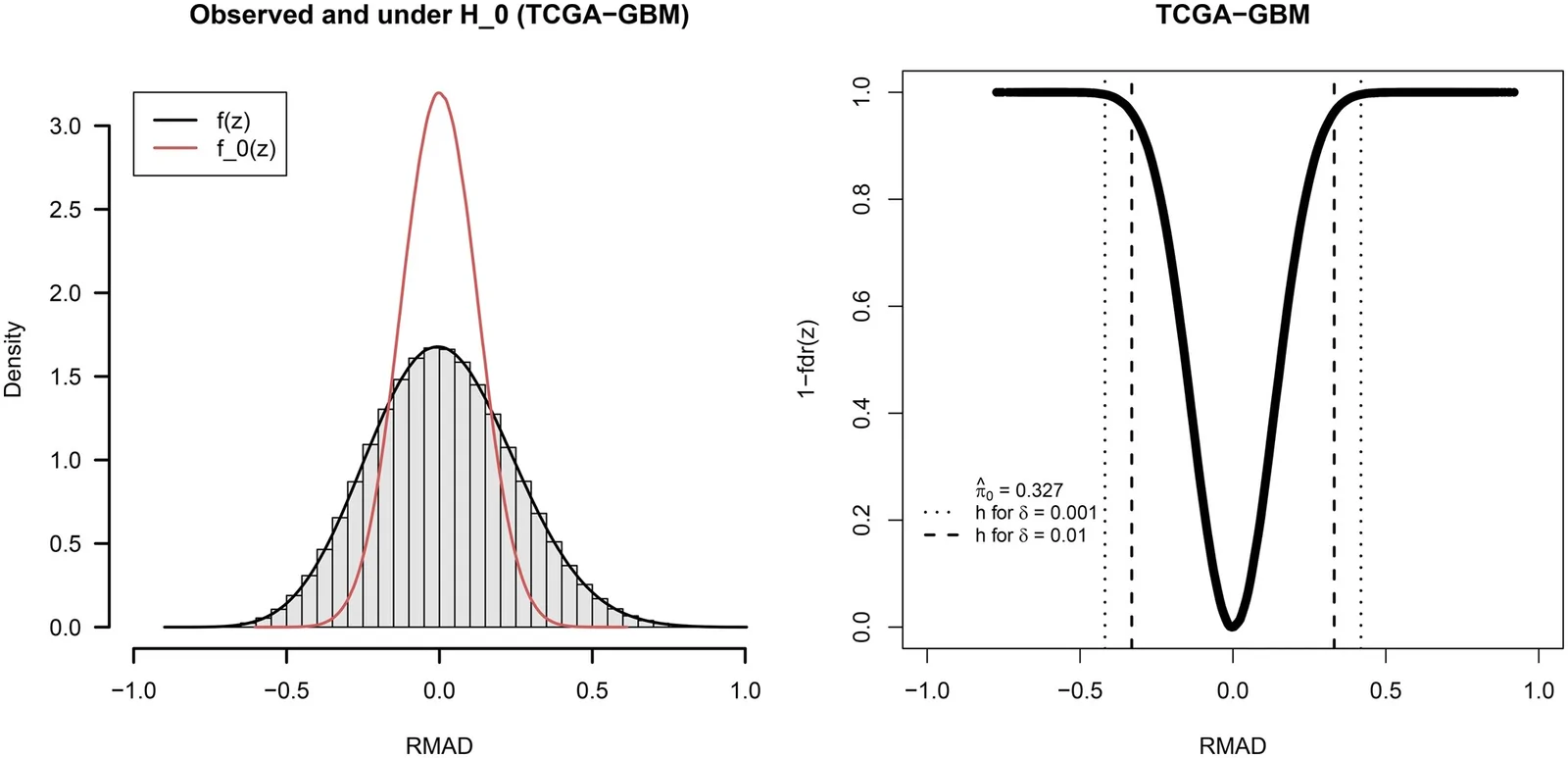
Results from the TCGA-GBM data using robust MAD correlation. Left panel: histogram and density of the observed correlations (f(z)), and the density of marginal correlations under the null ($f_{0}(z)$). Right panel: posterior probability of correlation 1−FDR(z) as a function of the observed correlations. The dotted and dashed vertical lines correspond to the threshold *h* when we control FDR at δ=0.001 and δ=0.01, respectively. The estimated proportion of null correlations is ${\hat{\pi }}_{0}=0.327$.

Using FDR control at 0.001, the analysis indicates that the threshold to create a sparse correlation matrix is at 0.4185, below which the correlations are set to zero. This threshold is lower than that in the glioma data. Looking at the distribution of f(z) and $f_{0}(z)$ in both datasets, the $f_{0}(z)$ in the TCGA-GBM data is tighter than that in the glioma data. This resulted in many more correlations with high posterior probability. At this threshold value, 92.68% of correlations are set to zero with 12.48 million correlations (7.32%) to be nonzero in the sparse correlation matrix. It can be shown that lowering the FDR (more stringent control) to 0.0001 means that the number nonzero correlations in the sparse matrix will be ∼6.33 million or 3.71%.

To illustrate the resulted sparse correlation matrix, Table 2 shows the number genes that are correlated with each of some known GBM genes (Liu *et al.* 2016, Yoon *et al.* 2023, Huang *et al.* 2024). The table shows that their networks are quite extensive with hundreds to thousands of connections. To further illustrate the gene ontology biological processes involved in those genes, Table 3 gives a list for gene NF1. Mutations or deletions in the NF1 gene, encoding neurofibromin 1, are associated with the mesenchymal subtype of GBM and contribute to tumour aggressiveness (Huang *et al.* 2024). Loss of NF1 disrupts apoptotic pathways, rendering GBM cells more resistant to programmed cell death and contributing to therapy resistance (D’Angelo *et al.* 2019). This gives relevant biological processes in Table 3 of those genes that are correlated with NF1.

**Table 2:** Number of genes that are correlated with each of GBM-related genes in the sparse correlation matrix in the TCGA-GBM data using robust MAD correlation and FDR control at 0.001.

TP53	IDH1	RAC3	PTEN	NF1
**440**	1025	2251	449	3843
**MGMT**	**EGFR**	**NDC80**	**ADAM22**	**SHANK1**
**1445**	1159	1228	2253	1933

**Table 3:** Gene ontology biological processes of genes that are correlated with NF1 as produced by PANTHER classification (Thomas *et al.* 2022).^a^

Biological process (NF1)	*P*-value
**Regulation of cell proliferation and tumour growth**	
**Reg. of cell cycle (GO:0051726)**	$9.43\times 10^{-6}$
**Reg. of cell cycle process (GO:0010564)**	$4.50\times 10^{-3}$
**Reg. of cellular component organization**	$1.54\times 10^{-8}$
** (GO:0051128)**	
**RAS/MAPK and intracellular signal transduction**	
**Small GTPase-mediated signal**	
** transduction (GO:0007264)**	$1.17\times 10^{-2}$
**Intracellular signal transduction**	
** (GO:0035556)**	$2.61\times 10^{-3}$
**Reg. of intracellular signal**	
** transduction (GO:1902531)**	$1.58\times 10^{-5}$
**DNA damage response and repair**	
**Regulation of DNA repair (GO:0006282)**	$7.48\times 10^{-8}$
**Positive regulation of**	
**DNA repair (GO:0045739)**	$4.42\times 10^{-5}$
**Positive reg. of double strand**	
** break repair (GO:2000781)**	$2.46\times 10^{-4}$
**Apoptosis and cell death**	
**Apoptotic process (GO:0006915)**	$1.71\times 10^{-5}$
**Programmed cell death (GO:0012501)**	$8.90\times 10^{-5}$
**Cell death (GO:0008219)**	$1.26\times 10^{-4}$

a The *P*-value is from over-representation test, with Bonferroni multiplicity correction. Not all biological processes are presented in the table—the purpose of this part of table is to illustrate some biological processes involved among genes that are correlated with NF1 in the sparse correlation matrix.

## 4 Discussion

Our contribution in this study is in the use of FDR control when we threshold the correlations in creating a sparse correlation matrix. In addition to the calculation of the correlation itself, thresholding is a critical step to decide which correlations are set to zero. Our approach provides a sensible interpretation in the resulting sparse correlation matrix, where we have an inference statement on how many (nonzero) correlations are expected to be false positives. In essence, we consider the creation of a sparse correlation matrix in the context of inference on the correlation.

Our procedure is best regarded as an empirical-Bayes calibration of the hard-thresholding step, rather than as an exact finite-sample multiple-testing guarantee. The identity FDR(R)=E[fdr(Z)|Z∈R] follows from the two-group mixture formulation and therefore does not, in itself, require independence among the pairwise correlation statistics. However, the practical accuracy of the resulting FDR estimates depends on reliable estimation of the mixture components f(z), $f_{0}(z)$, and $\pi_{0}$. In large-scale testing, estimation of $\pi_{0}$ can be conservative when a substantial fraction of non-null effects are weak and therefore insufficiently separated from the null component, which in turn inflates the estimated FDR and reduces power (Pawitan *et al.* 2005). Moreover, under general dependence, correlation among test statistics may leave the average FDR approximately unbiased while substantially increasing the variability of the realized FDP (Pawitan *et al.* 2006). These considerations are particularly relevant here because pairwise correlations are intrinsically dependent: many statistics share one or both genes. Accordingly, the reported FDR should be interpreted as a model-based, marginal estimate for the retained correlations, rather than as an exact guarantee under arbitrary dependence. Consistent with this interpretation, the permutation scheme used to estimate $f_{0}(z)$ is intended to calibrate the one-dimensional null distribution of the pairwise correlation statistic, not to reproduce the full joint dependence structure of the entire correlation matrix; more dependence-aware alternatives may be considered when preservation of broader null correlation structure is of primary interest (Pawitan *et al.* 2006). In addition, estimation error in $f_{0}(z)$ or $\pi_{0}$ propagates directly to the local FDR values and hence to the selected threshold: underestimation may render the procedure anticonservative, whereas overestimation leads to a more conservative threshold. These limitations are most acute in smaller datasets or when the null and alternative components are only weakly separated.

Our simulation study indicates that, when hard thresholding is used, selecting the threshold by minimizing the FN can materially reduce sensitivity for detecting true correlations, especially when those correlations are moderate or weak. This does not imply that hard thresholding is inappropriate, nor that Frobenius-norm minimization fails to control FDR; rather, it reflects a difference in objective between minimizing overall matrix-estimation error and retaining truly associated gene pairs under an explicit bound on the expected proportion of false discoveries. It is also useful to distinguish this inferential target from the simulation summaries used to assess it: the proposed threshold is calibrated by the marginal FDR, namely the expected proportion of false discoveries among retained correlations (Benjamini and Hochberg 1995, Genovese and Wasserman 2002), whereas the realized FDP in any single dataset is a random quantity that may vary substantially across replicates, particularly under dependence or marked sparsity (Meinshausen and Bühlmann 2006, Pawitan *et al.* 2006). For that reason, the simulation focuses on sensitivity, specificity, and FN as measures of recovery of the sparse correlation structure. Our approach enables more sensitivity to detect true correlations in such case, while maintaining high specificity and low FN. In the context of high correlation, then both approaches give similar results. However, in a general situation, the proportion of high correlations in the data is relatively very small while that of moderate correlations is quite substantial. With these characteristics, our approach is advantageous in addition to easier interpretation on the nonzero correlations in the sparse correlation matrix. In real-data applications, an additional practical benefit is that the retained network has a direct error-rate interpretation: at a specified value of δ, the expected proportion of falsely retained correlations is explicitly controlled, whereas a threshold selected by minimizing Frobenius loss provides no corresponding inferential statement for the retained edges. This data-adaptive interpretation supports transparent prioritization of high-confidence associations for downstream pathway analysis or experimental investigation, while less stringent values of δ can be used when broader coverage of moderate correlations is required.

The choice of the FDR control level also reflects the usual trade-off between sparsity and sensitivity. A more stringent value of δ yields a higher threshold *h* and a sparser matrix, but may remove moderate correlations; conversely, a more relaxed value of δ retains more correlations at the cost of allowing a higher expected proportion of false positives. This trade-off is not determined by the thresholding rule alone, but also by the proportion of truly correlated pairs, the distribution of the nonzero correlations, measurement variability, sample size, and dependence structure, factors that are known to govern FDR and sensitivity in high-dimensional genomic studies (Pawitan *et al.* 2005). In practice, we therefore recommend using stringent FDR control for an initial sparse and interpretable representation, followed by sensitivity analysis over a small range of δ values when moderate correlations are of scientific interest. More extreme small-sample regimes, including n<10, and count-data simulations are important extensions, but would introduce additional considerations—such as signal prevalence, measurement variability, library size, dispersion, zero counts, and normalization—that can themselves affect FDR, sensitivity, and the induced correlation structure (Pawitan *et al.* 2005, Law *et al.* 2014, Kruppa *et al.* 2016, Vandenbon 2022).

For gene-expression data, the principal considerations in selecting a correlation measure are robustness to sampling variation, outlying observations, and technical background variability, all of which can obscure biological association and distort correlation-based analyses, especially in high-dimensional settings (Klebanov and Yakovlev 2007, Serra *et al.* 2018, Moutsopoulos *et al.* 2021). We therefore recommend the robust MAD correlation for such data. We do not recommend, however, to use the Pearson correlation due to the danger of false positives (from biological perspective). It is well known that gene-wise variance can be underestimated by chance (Efron *et al.* 2001, Smyth 2004). Because they serve as denominator in the calculation of Pearson correlation, the problem of false positives (from biological perspective) is acute. The Pearson correlation is included in our computation tool simply for ‘completeness’ as it is a well-known method and, from statistical inference point of view, there is no problem in the implementation of the proposed method. As we discussed earlier, our approach in creating a sparse correlation matrix is not dependent on which correlation measure to use.

## 5 Conclusion

Creating a sparse correlation matrix is a critical task in genomic analysis, especially when the correlation matrix will be interpreted or subsequently be involved for further analysis. We propose a novel method in the thresholding to create a sparse correlation matrix by controlling the FDR of nonzero correlations. This provides a straightforward intuitive interpretation and shown to have a good operating characteristics in identifying true correlations.

## Supplementary Material

vbag314_Supplementary_Data

## References

[vbag314-B1] Barretina J , Caponigro G, Stransky N et al The Cancer Cell Line Encyclopedia enables predictive modelling of anticancer drug sensitivity. Nature 2012;483:603–7. 10.1038/nature11003 PMC3320027 22460905

[vbag314-B2] Benjamini Y , Hochberg Y. Controlling the false discovery rate: a practical and powerful approach to multiple testing. J. R. Stat. Soc. B 1995;57:289–300. 10.1111/j.2517-6161.1995.tb02031.x

[vbag314-B3] Bernal V , Bischoff R, Guryev V et al Exact hypothesis testing for shrinkage-based Gaussian graphical models. Bioinformatics 2019;35:5011–7. 10.1093/bioinformatics/btz357 PMC6901079 31077287

[vbag314-B4] Bickel PJ , Levina E. Covariance regularization by thresholding. Ann Statist 2008;36:2577–604. 10.1214/08-AOS600

[vbag314-B5] Bolstad BM , Irizarry RA, Astrand M et al A comparison of normalization methods for high density oligonucleotide array data based on bias and variance. Bioinformatics 2003;19:185–93. 10.1093/bioinformatics/19.2.185 12538238

[vbag314-B6] Brennan CW , Verhaak RGW, McKenna A et al; TCGA Research Network. The somatic genomic landscape of glioblastoma. Cell 2013;155:462–77. 10.1016/j.cell.2013.09.034 PMC3910500 24120142

[vbag314-B7] Cai TT , Liu W. Large-scale multiple testing of correlations. J Am Stat Assoc 2016;111:229–40. 10.1080/01621459.2014.999157 PMC4894362 27284211

[vbag314-B8] Cancer Genome Atlas Research Network. Comprehensive genomic characterization of squamous cell lung cancers. Nature 2012;489:519–25. 10.1038/nature11404 PMC3466113 22960745

[vbag314-B9] Cancer Genome Atlas Research Network. Comprehensive molecular profiling of lung adenocarcinoma. Nature 2014;511:543–50. 10.1038/nature13385 PMC4231481 25079552

[vbag314-B10] Colby SM , McClure RS, Overall CC et al Improving network inference algorithms using resampling methods. BMC Bioinformatics 2018;19:376. 10.1186/s12859-018-2402-0 PMC6186128 30314469

[vbag314-B11] D’Angelo F , Ceccarelli M, Garofano L et al The molecular landscape of glioma in patients with neurofibromatosis 1. Nat Med 2019;25:176–87. 10.1038/s41591-018-0263-8 PMC6857804 30531922

[vbag314-B12] Efron B , Tibshirani R, Storey JD et al Empirical Bayes analysis of a microarray experiment. J Am Stat Assoc 2001;96:1151–60. 10.1198/016214501753382129

[vbag314-B13] Friedman J , Hastie T, Tibshirani R et al Sparse inverse covariance estimation with the graphical lasso. Biostatistics 2008;9:432–41. 10.1093/biostatistics/kxm045 PMC3019769 18079126

[vbag314-B14] Gaiteri C , Ding Y, French B et al Beyond modules and hubs: the potential of gene coexpression networks for investigating molecular mechanisms of complex brain disorders. Genes Brain Behav 2014;13:13–24. 10.1111/gbb.12106 PMC3896950 24320616

[vbag314-B15] Garnett MJ , Edelman EJ, Heidorn SJ et al Systematic identification of genomic markers of drug sensitivity in cancer cells. Nature 2012;483:570–5. 10.1038/nature11005 PMC3349233 22460902

[vbag314-B16] Genovese C , Wasserman L. Operating characteristics and extensions of the false discovery rate procedure. J. R. Stat. Soc.B 2002;64:499–517. 10.1111/1467-9868.00347

[vbag314-B17] Goldman MJ , Craft B, Hastie M et al Visualizing and interpreting cancer genomics data via the Xena platform. Nat Biotechnol 2020;38:675–8. 10.1038/s41587-020-0546-8 PMC7386072 32444850

[vbag314-B18] Grossman RL , Heath AP, Ferretti V et al Toward a shared vision for cancer genomic data. N Engl J Med 2016;375:1109–12. 10.1056/NEJMp1607591 PMC6309165 27653561

[vbag314-B19] Higham NJ. Computing the nearest correlation matrix—a problem from finance. IMA J Numer Anal 2002;22:329–43. 10.1093/imanum/22.3.329

[vbag314-B20] Huang Y-F , Chiao M-T, Hsiao T-H et al Genetic mutation patterns among glioblastoma patients in the Taiwanese population—insights from a single institution retrospective study. Cancer Gene Ther 2024;31:894–903. 10.1038/s41417-024-00746-y PMC11192627 38418842

[vbag314-B21] Irizarry RA , Bolstad BM, Collin F et al Summaries of Affymetrix GeneChip probe level data. Nucleic Acids Res 2003;31:e15. 10.1093/nar/gng015 PMC150247 12582260

[vbag314-B22] Jiang D , Tang C, Zhang A. Cluster analysis for gene expression data: a survey. IEEE Trans Knowl Data Eng 2004;16:1370–86. 10.1109/TKDE.2004.68

[vbag314-B23] Jiang B. Covariance selection by thresholding the sample correlation matrix. Stat Probab Lett 2013;83:2492–8. 10.1016/j.spl.2013.07.008

[vbag314-B24] Klebanov L , Yakovlev A. How high is the level of technical noise in microarray data? Biol Direct 2007;2:9. 10.1186/1745-6150-2-9 PMC1855048 17428341

[vbag314-B25] Kruppa J , Kramer F, Beißbarth T et al A simulation framework for correlated count data of features subsets in high-throughput sequencing or proteomics experiments. Stat Appl Genet Mol Biol 2016;15:401–14. 10.1515/sagmb-2015-0082 27655448

[vbag314-B26] Langfelder P , Horvath S. WGCNA: an R package for weighted correlation network analysis. BMC Bioinformatics 2008;9:559. 10.1186/1471-2105-9-559 PMC2631488 19114008

[vbag314-B27] Law CW , Chen Y, Shi W et al Voom: precision weights unlock linear model analysis tools for RNA-seq read counts. Genome Biol 2014;15:R29. 10.1186/gb-2014-15-2-r29 PMC4053721 24485249

[vbag314-B28] Liu A , Hou C, Chen H et al Genetics and epigenetics of glioblastoma: applications and overall incidence of IDH1 mutation. Front. Oncol 2016;6:16. 10.3389/fonc.2016.00016 PMC4731485 26858939

[vbag314-B29] Luo F , Yang Y, Zhong J et al Constructing gene co-expression networks and predicting functions of unknown genes by random matrix theory. BMC Bioinformatics 2007;8:299. 10.1186/1471-2105-8-299 PMC2212665 17697349

[vbag314-B30] Ma S , Shi M, Li Y et al Incorporating gene co-expression network in identification of cancer prognosis markers. BMC Bioinformatics 2010;11:271. 10.1186/1471-2105-11-271 PMC2881088 20487548

[vbag314-B31] McClintick JN , Edenberg HJ. Effects of filtering by present call on analysis of microarray experiments. BMC Bioinformatics 2006;7:49. 10.1186/1471-2105-7-49 PMC1409797 16448562

[vbag314-B32] Meinshausen N , Bühlmann P. High-dimensional graphs and variable selection with the Lasso. Ann Statist 2006;34:1436–62. 10.1214/009053606000000281

[vbag314-B33] Moutsopoulos I , Maischak L, Lauzikaite E et al noisyR: enhancing biological signal in sequencing datasets by characterizing random technical noise. Nucleic Acids Res 2021;49:e83. 10.1093/nar/gkab433 PMC8373073 34076236

[vbag314-B34] Paliwal KK , Sharma A. Improved direct LDA and its application to DNA microarray gene expression data. Pattern Recognit Lett 2010;31:2489–92. 10.1016/j.patrec.2010.08.003

[vbag314-B35] Pasman V , Shevlyakov G. Robust methods of estimation of correlation-coefficient. Autom. Remote Control 1987;48:332–40.

[vbag314-B36] Pawitan Y , Murthy KRK, Michiels S et al Bias in the estimation of false discovery rate in microarray studies. Bioinformatics 2005;21:3865–72. 10.1093/bioinformatics/bti626 16105901

[vbag314-B37] Pawitan Y , Calza S, Ploner A et al Estimation of false discovery proportion under general dependence. Bioinformatics 2006;22:3025–31. 10.1093/bioinformatics/btl527 17046978

[vbag314-B38] Pawitan Y , Michiels S, Koscielny S et al False discovery rate, sensitivity and sample size for microarray studies. Bioinformatics 2005;21:3017–24. 10.1093/bioinformatics/bti448 15840707

[vbag314-B39] Ploner A , Calza S, Gusnanto A et al Multidimensional local false discovery rate for microarray studies. Bioinformatics 2006;22:556–65. 10.1093/bioinformatics/btk013 16368770

[vbag314-B40] Rothman AJ , Bickel PJ, Levina E et al Sparse permutation invariant covariance estimation. Electron J Stat 2008;2:494–515. 10.1214/08-EJS176

[vbag314-B41] Rothman AJ , Levina E, Zhu J et al Generalized thresholding of large covariance matrices. J Am Stat Assoc 2009;104:177–86. 10.1198/jasa.2009.0101

[vbag314-B42] Serra A , Coretto P, Fratello M et al Robust and sparse correlation matrix estimation for the analysis of high-dimensional genomics data. Bioinformatics 2018;34:625–34. 10.1093/bioinformatics/btx642 29040390

[vbag314-B43] Shi H , Zhang L, Qu Y et al Prognostic genes of breast cancer revealed by gene co-expression network analysis. Oncol Lett 2017;14:4535–42. 10.3892/ol.2017.6779 PMC5649579 29085450

[vbag314-B44] Smyth GK. Linear models and empirical Bayes methods for assessing differential expression in microarray experiments. Stat Appl Genet Mol Biol 2004;3:1–25. 10.2202/1544-6115.1027 16646809

[vbag314-B45] Thomas PD , Ebert D, Muruganujan A et al PANTHER: making genome-scale phylogenetics accessible to all. Protein Sci 2022;31:8–22. 10.1002/pro.4218 PMC8740835 34717010

[vbag314-B46] Vandenbon A. Evaluation of critical data processing steps for reliable prediction of gene co-expression from large collections of RNA-seq data. PLoS One 2022;17:e0263344. 10.1371/journal.pone.0263344 PMC8797241 35089979

[vbag314-B47] Voigt A , Almaas E. Assessment of weighted topological overlap (wTO) to improve fidelity of gene co-expression networks. BMC Bioinformatics 2019;20:58. 10.1186/s12859-019-2596-9 PMC6350380 30691386

[vbag314-B48] Werhli AV , Husmeier D. Reconstructing gene regulatory networks with Bayesian networks by combining expression data with multiple sources of prior knowledge. Stat Appl Genet Mol Biol 2007;6:Article15. 10.2202/1544-6115.1282 17542777

[vbag314-B49] Yang Y , Han L, Yuan Y et al Gene co-expression network analysis reveals common system-level properties of prognostic genes across cancer types. Nat Commun 2014;5:3231. 10.1038/ncomms4231 PMC3951205 24488081

[vbag314-B50] Yoon HG , Cheong JH, Ryu JI et al The genes significantly associated with an improved prognosis and long-term survival of glioblastoma. PLoS One 2023;18:e0295061. 10.1371/journal.pone.0295061 PMC10686432 38019838

[vbag314-B51] Zhang B , Horvath S. A general framework for weighted gene co-expression network analysis. Stat Appl Genet Mol Biol 2005;4:Article17. 10.2202/1544-6115.1128 16646834

[vbag314-B52] Zhou H-Y , Wang Y-C, Wang T et al CCNA2 and NEK2 regulate glioblastoma progression by targeting the cell cycle. Oncol Lett 2024;27:206. 10.3892/ol.2024.14339 PMC10956385 38516683

