# Supplementary material for: False discovery rate control for sparse correlation matrix
Source: Bioinform Adv. 2026 Oct 19;6(1):vbag314. doi: 10.1093/bioadv/vbag314 (PMC13744407; doi:10.1093/bioadv/vbag314)
Supplement: Supplementary file 1 [file vbag314_supplementary_data.pdf]

Supplementary material

for the manuscript

False Discovery Rate Control for Sparse Correlation Matrix

Arief Gusnanto[a*], Basuki Widodo[b], Norli Anida Abdullah[c], Ahmad Zia Ul-Saufie[d], Lucy F. Stead[e]

[a]Department of Statistics, University of Leeds, Leeds LS2 9JT, United Kingdom
[b]Department of Mathematics, Institut Teknologi Sepuluh Nopember, Surabaya 60111, Indonesia
[c]Mathematics Division, Centre for Foundation Studies in Science, Universiti Malaya, Kuala Lumpur 50603, Malaysia
[d]Faculty of Computer and Mathematical Sciences, Universiti Teknologi MARA, Shah Alam 40450, Malaysia
[e]Leeds Institute of Medical Research at St. James's, University of Leeds, Leeds LS9 7TF, United Kingdom.

In this supplementary material, we present some additional figures related to the results of analysis. The mathematical notation in this document follows that in the main text.

Estimating the threshold h to create sparse correlation matrix.

With p genes in the data, we have $n_q = p(p-1)/2$ observed pairwise correlations between gene j and j', denoted $z_{jj'}$, $j, j' \in \{1, 2, \ldots, p\}$ and $j < j'$. For the purpose of estimating the threshold h in this section, we replace the index of z into q with $q = 1, 2, \ldots, n_q$. Let $\widehat{\text{fdr}}(z_q)$ be the estimated local false discovery rate associated with z_q. For a threshold $h \geq 0$, we define the selected set

$$\mathfrak{R}(h) = \{q : |z_q| \geq h\},$$

where $|z_q|$ denotes the absolute value of z_q. This corresponds to retaining all observed correlations whose absolute values exceed h, and setting the remainder to zero in the sparse correlation matrix.

Let

$$N(h) = |\mathfrak{R}(h)|$$

denote the number of retained correlations at threshold h. The estimated global FDR at threshold h is

$$\widehat{\text{FDR}}(h) = \frac{1}{N(h)} \sum_{q:|z_q|\geq h} \widehat{\text{fdr}}(z_q),\ N(h) > 0.$$

In practice, we do not usually search over all possible threshold directly. Instead, we rank the observed correlations by decreasing absolute value:

$$|z|_{(1)} \geq |z|_{(2)} \geq \ldots \geq |z|_{(n_q)}.$$

For the top t correlations, the estimated global FDR is given by

$$\widehat{\text{FDR}}(t) = \frac{1}{t} \sum_{q=1}^{t} \widehat{\text{fdr}}(z_{(q)}),$$

and is evaluated at $t = 1, 2, \ldots, n_q$. This is the running average of the local fdr over the top t observed correlations ranked by $|z|$. The threshold h is chosen as

$$h = |z|_{t^*},$$

where

$$t^* = \max\left\{t : \widehat{\text{FDR}}(t) \leq \delta\right\}.$$

*Contact: Arief Gusnanto, Email: a.gusnanto@leeds.ac.uk

Simulation results using Spearman rank correlation

ρ	FDR	$\Psi_{kk} : 50 \times 50$			$\Psi_{kk} : 25 \times 25$		
		Sens.	Spec.	FN	Sens.	Spec.	FN
0.3	RSC	0.069	1.000	0.000	0.016	1.000	0.000
	0.001	0.211	1.000	0.000	0.198	1.000	0.000
	0.002	0.280	0.999	0.000	0.260	1.000	0.000
	0.003	0.326	0.999	0.000	0.303	0.999	0.000
	0.005	0.388	0.998	0.000	0.362	0.999	0.000
	0.010	0.481	0.997	0.001	0.456	0.997	0.000
	0.015	0.540	0.995	0.001	0.518	0.995	0.000
	0.020	0.583	0.993	0.001	0.563	0.994	0.001
	0.050	0.723	0.979	0.002	0.712	0.980	0.001
0.5	RSC	0.900	1.000	0.000	0.832	1.000	0.000
	0.001	0.958	0.999	0.000	0.943	1.000	0.000
	0.002	0.971	0.998	0.000	0.959	0.999	0.000
	0.003	0.977	0.998	0.000	0.967	0.999	0.000
	0.005	0.983	0.997	0.000	0.976	0.998	0.000
	0.010	0.989	0.995	0.001	0.985	0.996	0.000
	0.015	0.992	0.992	0.001	0.990	0.995	0.001
	0.020	0.994	0.990	0.001	0.992	0.993	0.001
	0.050	0.998	0.976	0.002	0.997	0.980	0.001
0.7	RSC	1.000	1.000	0.000	0.999	1.000	0.000
	0.001	1.000	0.999	0.000	1.000	0.999	0.000
	0.002	1.000	0.998	0.000	1.000	0.999	0.000
	0.003	1.000	0.998	0.000	1.000	0.999	0.000
	0.005	1.000	0.997	0.000	1.000	0.998	0.000
	0.010	1.000	0.994	0.001	1.000	0.996	0.000
	0.015	1.000	0.992	0.001	1.000	0.995	0.001
	0.020	1.000	0.990	0.001	1.000	0.993	0.001
	0.050	1.000	0.976	0.002	1.000	0.980	0.001
0.9	RSC	1.000	1.000	0.000	1.000	1.000	0.000
	0.001	1.000	0.999	0.000	1.000	0.999	0.000
	0.002	1.000	0.998	0.000	1.000	0.999	0.000
	0.003	1.000	0.998	0.000	1.000	0.999	0.000
	0.005	1.000	0.997	0.000	1.000	0.998	0.000
	0.010	1.000	0.994	0.001	1.000	0.996	0.000
	0.015	1.000	0.992	0.001	1.000	0.995	0.001
	0.020	1.000	0.990	0.001	1.000	0.993	0.001
	0.050	1.000	0.976	0.002	1.000	0.980	0.001

Table S1: Sensitivity, specificity, and Frobenius norm (FN) of sparse correlation matrix from 1000 simulated data under different simulation settings using the Spearman rank correlation. The FDR is controlled from 0.001 to 0.05, with the 'RSC' row corresponds to construction of sparse correlation matrix by minimising the Frobenius norm (Serra *et al.*, 2018). The true correlation ρ is set to be from 0.3-0.9, and the dimension of the Ψ_{11} and Ψ_{22} sub-matrix in the true correlation matrix is 50×50 and 25×25 (Eq. (9) in the main text). The simulation results using the robust MAD correlation are presented in Table 1 in the main text.

Glioma data (GSE36133): circular plots

We present additional figures to illustrate some correlations from the sparse correlation matrix (with FDR 0.001, using robust MAD correlation) in the main analysis. We draw a circular plot for genes that are correlated with each of NEK2 and CCNA2 as shown in Figure S1. Both genes are recently been identified to regulate glioblastoma progression by targeting the cell cycle (Zhou *et al.*, 2024). In the case of NEK2, the gene is correlated with 16 other genes while in the case of CCNA2, it is correlated with 42 other genes.

These circular plots are intended as illustrative summaries rather than exhaustive displays of overlap; in particular, for genes represented by multiple probesets, incomplete concordance may arise both from stringent thresholding and from differences in the transcript regions targeted by the probesets.

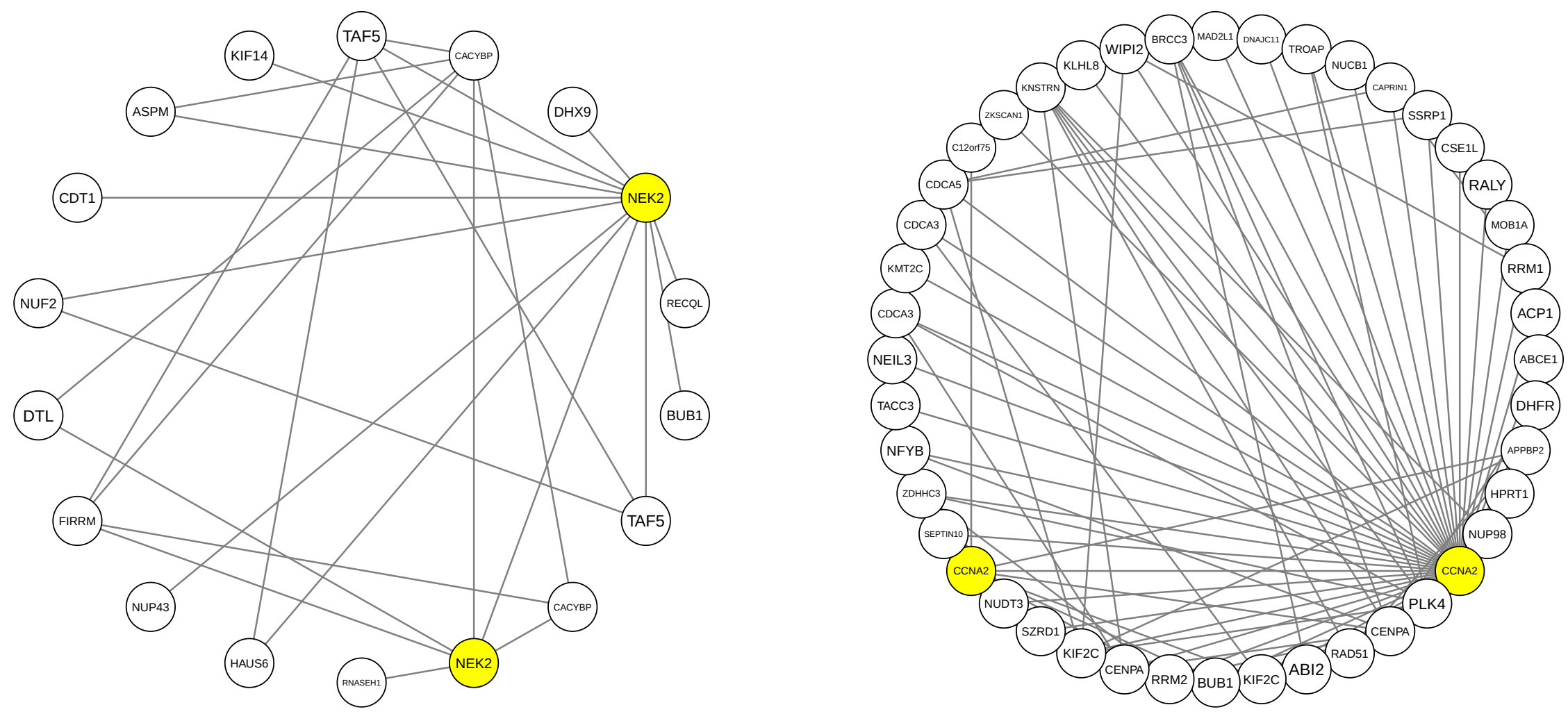

Figure S1: *Genes that are declared to be correlated after thresholding of robust MAD correlations in the glioma data for NEK2 (left panel) and CCNA2 (right panel). Each of NEK2 and CCNA2 is represented by two probesets.*

Glioma data (GSE36133): Spearman rank correlation

In this section, we present the results of analysis on the Glioma data when we consider the Spearman rank correlation. This is to illustrate the generalisability of the proposed methodology to use other correlation measures.

Figure S2 presents the results on analysis of the glioma data (GSE36133) using the Spearman correlation. The figure indicates similar conclusion to that from Figure 1 in the main paper. The histogram of the observed Spearman correlation (Figure S2 left) indicates less variance (mean(z)=0.0192, sd(z)=0.1990, median(z)=0.0174) than the robust MAD correlations in Figure 1 in the main paper. The middle panel of Figure S2 shows the kernel densities of $f(z)$ and $f_0(z)$. The figure indicates a slight excess of variability of $f(z)$ compared to $f_0(z)$. As a result of this, Figure S2 (right panel) shows the posterior probability of correlation as a function of the observed Spearman correlations.

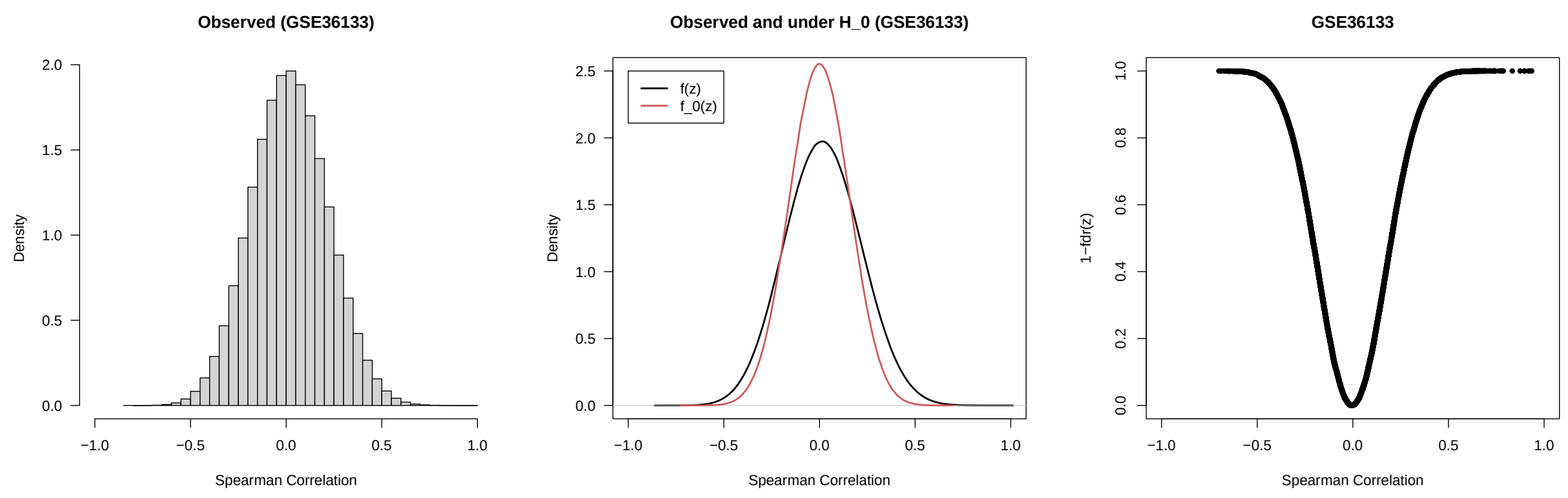

Figure S2: *Results from the glioma data (GSE36133) using Spearman correlation: Histogram of observed correlations (left), density of the observed ($f(z)$) and null ($f_0(z)$) correlations (middle), and posterior probability of correlation* $1 - fdr(z)$ *(right).*

The estimated threshold for controlling FDR at 0.001 is 0.561. As a result of this threshold, there are approximately 84.7 million correlations (or 99.6%) set to zero and approximately 340,000 (≈0.4%) correlations to be non-zero (above the threshold).

TCGA GBM data: Spearman rank correlation

For the TCGA-GBM data, Figure S3 presents the results using the Spearman correlation. The distribution of the observed Spearman correlations is shown in the left panel (mean(z)=0.0103, sd(z)=0.2090, median(z)=0.0040). The standard deviation is slightly smaller than the standard deviation of robust MAD correlations in Figure 3 of the main paper. The middle figure shows the density of the observed Spearman correlation, $f(z)$, in comparison to that under the null hypothesis that there is no correlation between genes, $f_0(z)$. The right panel shows the posterior probability of correlation $1 - \text{fdr}(z)$.

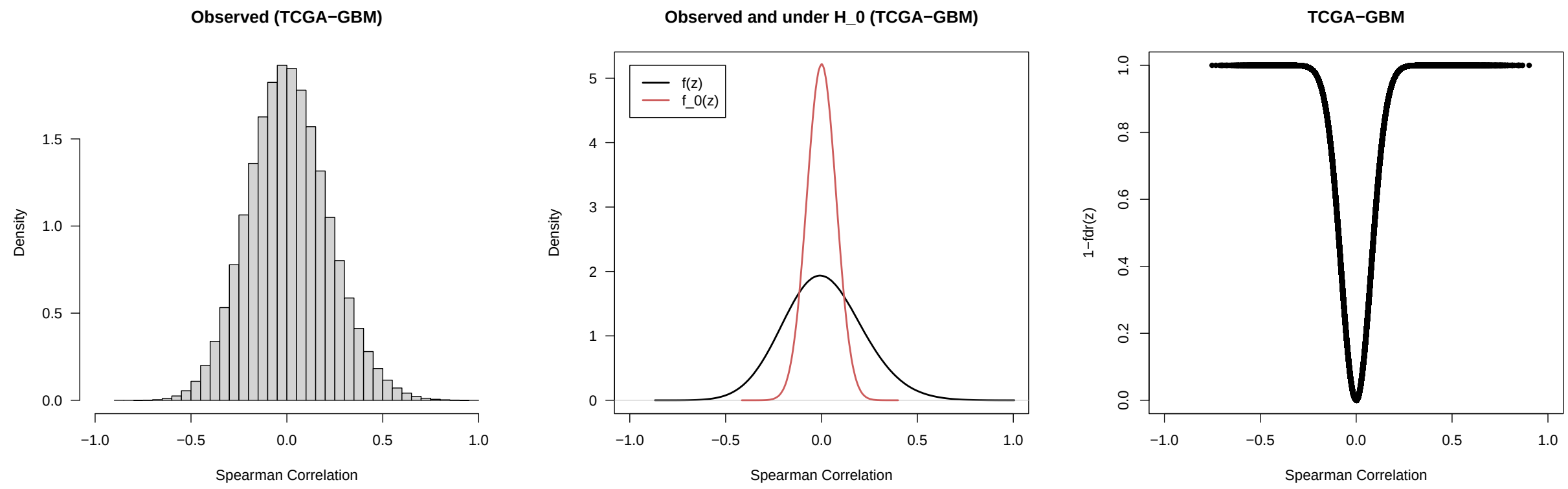

Figure S3: *Results from the TCGA-GBM data using Spearman correlation: Histogram of observed correlations (left), density of the observed ($f(z)$) and null ($f_0(z)$) correlations (middle), and posterior probability of correlation $1 - fdr(z)$ (right).*

Controlling for FDR at 0.001, the threshold identified is at 0.247. At this threshold, the number of correlations that are set to zero is approximately 130.4 million or 76.5%, while approximately 40.1 million or 23.5% correlations are non zero. Looking at the distribution of $f_0(z)$ in Figure S3 (middle panel), it is quite concentrated. As a result, a medium Spearman correlation in the TCGA-GBM data would have a relatively low local false discovery rate (Figure S3 right panel). When we control the FDR at 0.0001 (more stringent), the threshold is at 0.294. At this threshold, the number of zero correlations is at 84.2% while the non-zero correlations is at 15.8%, approximately.

References

Serra, A, *et al.* (2018), Robust and sparse correlation matrix estimation for the analysis of high-dimensional genomics data, *Bioinformatics*, **34**(4):625-634

Zhou, HY *et al.* (2024) CCNA2 and NEK2 regulate glioblastoma progression by targeting the cell cycle. *Oncology Letters*, **27**(5):206.
